# Electroencephalographic assessment of pneumatic penetrating captive-bolt stunning of donkeys *(Equus asinus)*

**DOI:** 10.1017/awf.2026.10090

**Published:** 2026-05-28

**Authors:** Katharine Ann Fletcher, Beatrice Benedetti, Barbara Padalino, Mariano Hernández Gil, Troy Gibson

**Affiliations:** 1 Royal Veterinary College Department of Pathology and Infectious Diseases, United Kingdom; 2 University of Bologna, Italy; 3 University of Bologna Department of Agricultural Sciences, Italy; 4 Southern Cross University, Australia; 5 National Autonomous University of Mexico, Mexico; 6 Royal Veterinary College, United Kingdom

**Keywords:** Animal welfare, captive-bolt device, donkeys, electroencephalography, slaughter, stunning

## Abstract

Equids slaughtered for human consumption in many countries are stunned with penetrating captive bolt (PCB) to produce an irreversible state of unconsciousness to prevent pain and distress before exsanguination. This topic is much-studied for the most commonly slaughtered species, while literature remains limited on Equidae, particularly donkeys (*Equus asinus*). This study, in a Mexican commercial abattoir, examined the effectiveness of pneumatic PCB stunning with electroencephalographic (EEG) and behavioural signs in 13 donkeys. Forty-six percent (6/13) of donkeys had periods of ‘normal-like’ EEG after PCB stunning, between 1 to 9 s in duration. However, in all animals, this changed to either ‘transitional’ EEG or ‘isoelectric’ waveforms. The normal-like EEG phases were characterised by increased theta, alpha and beta activity in the EEG power spectrum. Just one donkey showed behavioural signs of incomplete concussion after stunning, showing both rhythmic breathing and spontaneous blinking, alongside normal-like EEG. In addition, four animals did not reach isoelectric EEG during the 30 s of recording. Shot position frequently deviated caudally from the suggested position and this was significantly associated with the presence of normal-like EEG and behavioural signs. These results highlight welfare concerns related to delayed or incomplete loss of consciousness in these animals, indicating the need for species-specific stunning guidelines. In conclusion, EEG is a useful research tool to substantially assist in understanding the risks of a potential return of consciousness after stunning. This could help to refine ideal airline pressure, shot position and validate behavioural signs that better evaluate consciousness in donkeys.

## Introduction

Donkeys ( *Equus asinus* ) are slaughtered across the world for a variety of reasons, including their meat and by-products, particularly their skins, which are used to make ‘eijao’, a product used in Traditional Chinese Medicine (Gameiro *et al.* 2021; Norris *et al.* 2021). Despite increasing rates of donkey slaughter (Bennett & Pfuderer 2019; Gameiro *et al.* 2021), very little research has been conducted into the methods used or the factors affecting their effectiveness and the impact on welfare (Fletcher *et al.* 2022, 2024).

Penetrating Captive Bolt (PCB) is one of the most common methods used for the stunning of livestock prior to slaughter (Oliveira *et al.* 2018). Unconsciousness is induced through the combination of the transference of the kinetic energy of the moving metal bolt to the cranium and brain, and the direct physical trauma to critical brain structures (Terlouw *et al.* 2016; Baier & Willson 2020). The extent of focal and diffuse damage to brain structures depends largely on placement and orientation of the bolt into the brain (Terlouw *et al.* 2016). The highly referred to positioning for PCB slaughter of horses is 10 mm above the intersection of two imaginary lines drawn from the centre middle of the ears and the inside of the eye on the opposite side (Humane Slaughter Association 2016). No such guidance exists for donkeys, however anecdotally most operators tend to use the same position (K Fletcher, personal communication and prior field observation 2023, 2024).

Efficacy and the welfare of animals during PCB stunning has been examined via a variety of indices, including behaviour observations (Verhoeven *et al.* 2015; Terlouw 2020; Fletcher *et al.* 2024), changes in the electroencephalogram (EEG) (Verhoeven *et al.* 2015; Gibson *et al.* 2019; Dalla Costa *et al.* 2020), evoked potentials (Verhoeven *et al.* 2015) and trauma to associated brain structures (Dalla Costa *et al.* 2020). Unlike behavioural and brainstem indices of brain activity, the EEG represents the direct functional activity of the brain and is considered a more reliable indicator of when undoubted unconsciousness occurs (Gibson *et al.* 2019).

EEG provides the real-time functional activity of the brain and can be used to complement indirect behavioural indicators after stunning at slaughter (Verhoeven *et al.* 2015; Gibson *et al.* 2019; Dalla Costa *et al.* 2020; Kumar *et al.* 2023). As the level of unconsciousness deepens, there is a reduction in total power (Ptot) EEG (Verhoeven *et al.* 2015) and/or a frequency shift to a predominance of low frequency activity. A conscious animal showing ‘normal-like’ (or ‘baseline’) EEG will typically display a combination of low, mid and high frequency activity, with alpha (8 to 12 Hz) waves indicating relaxation and beta (12–30 Hz) indicating a stressed or fearful state (Verhoeven *et al.* 2015; Terlouw *et al.* 2016; Kumar *et al.* 2023). In TBI-induced unconsciousness, animals generally display predominantly high-amplitude, low-frequency ‘transitional’ delta (0.5 to 4 Hz) and theta (4 to 8 Hz) with an absence of alpha or beta activity. These can be recoverable, but an animal is considered to have irrecoverable unconsciousness, or brain death, when an isoelectric waveform is present (Verhoeven *et al.* 2015; Terlouw *et al.* 2016; Grandin 2020; Kumar *et al.* 2023).

Donkeys are considered stoic and subtle in their behavioural display (Ashley *et al.* 2005), which could lead to misunderstanding or misinterpretation when being assessed during the slaughter process. Therefore, in a research context, the combination of EEG and behavioural indices could provide a more reliable, robust and accurate assessment of welfare and stun effectiveness compared to assessment of behaviour alone. EEG has been successfully used to measure and validate stunning efficiency in other animals at slaughter (Zulkifli *et al.* 2014; Verhoeven *et al.* 2016; Gibson *et al.* 2019). However, to date, there have been no studies that have used EEG activity to evaluate the loss of consciousness of donkeys at slaughter.

The aim of this study was to examine the EEG responses of commercially slaughtered donkeys to PCB stunning and determine how these responses were related to assessed behavioural/brainstem reflexes.

## Materials and methods

The study was carried out over six days in April 2024, during routine commercial stunning and slaughter of donkeys (n = 13) at a Mexican abattoir. This project had ethical approval from the Royal Veterinary College Clinical Research Ethical Review Board (URN 2024 2268-3).

Donkeys arrived at the abattoir either on the same day or the day before slaughter. Information about the sex, age, background/origin of the animals and the transport length was not available due to restricted information at the abattoir. The donkeys were slaughtered in the same slaughter line and stun box as the horses and cattle. All animals entered a standard single-file straight raceway and were then individually led, through a manually opened gate, into a cattle stun box. Animals were stunned by multiple commercial operators; it was not possible to measure operator effect due to sensitivity within the abattoir. Convenience sampling was used based on order of animals throughout the day and was dependent upon permission/consent granted by the abattoir and/or suppliers. Prior to, and during, stunning, donkeys were physically restrained with a head/neck rope held by an operator to allow for placement of EEG electrodes for pre-treatment recording. Instrumentation took between 10–20 s, followed by a minimum of 30 s of pre-treatment EEG recorded. Traces were monitored to ensure at least 20 s of continuous stable EEG recorded prior to the shot. After pre-treatment recording, donkeys were shot in a frontal position with a Jarvis USSS-1 penetrating pneumatic stunner (USSS-1, JARVIS® Jarvis Products Corporation; Middletown, CT, USA). Penetrating captive-bolt airline pressure and bolt velocity could not be recorded as there were no in-line meters/regulators. The compressor tank air pressure was set to 120 psi (827 kPa). One minute after the shot, donkeys were released, via a second manually operated gate, into the bleeding area. Prior to bleeding, each donkey was ear-tagged to allow head identification, post mortem.

### Behavioural assessment

Immediately after shooting, each donkey was assessed for signs of effectiveness of stunning by three members of the research team from different positions (side of the stun box, front of the stun box, and in the roll-out area). All three observers remained consistent in their individual positions, assessing animals at different points with constant communication between the team to confirm observations. In particular, the presence or absence of immediate collapse, rhythmic breathing, palpebral and corneal reflex, spontaneous eye blinking, eyeball rotation, nystagmus, gasping, response to stimulation of the septum (with forceps), tonic and clonic convulsions were evaluated after the PCB shot until shackling and hoisting for exsanguination (Gibson *et al.* 2015; Fletcher *et al.* 2024) (Table 1).

**Table 1.** Brainstem and behavioural signs of consciousness assessed immediately post pneumatic penetrating captive bolt (PCB) shot in donkeys (n = 13) in a Mexican abattoir (adapted from Gibson *et al.* 2015) Table 1. long description.

Behaviours cranial/spinal responses	Description
Lack of immediate collapse	Animal fails to collapse immediately after the shot
Righting reflex	Makes co-ordinated effort to stand or lift head
Vocalisations	Vocalises independently from exhalation
Rhythmic respiration	Ribcage continuously moves in and out rhythmically
Gasping	Spasmodic sharp intake of breath with mouth open
Corneal reflex	Involuntary blink reflex when cornea is stimulated
Palpebral reflex	Involuntary blink reflex when the medial canthus is stimulated
Spontaneous blinking	Opens/closes eyelid without stimulation
Nystagmus	Rapid involuntary movements of the eye
Eyeball rotation	Eyes rotated, not central, sclera visible
Response to nostril pinch	Pinching nasal septum is followed by pain reaction/ withdrawal
Involuntary muscle spasms	Absence of tonus in body/excessive muscle activity

Donkeys were classified as incompletely concussed if they failed to collapse or rhythmic breathing was present and/or if at least two of the following parameters were present: positive corneal reflex; positive palpebral reflex; eyeball rotation; and nystagmus (Gibson *et al.* 2015). Behaviours and brainstem indices were sampled continuously from immediately after the shot (including the EEG recording period, EEG electrode removal and roll-out), with observations continuing until animals were shackled and hoisted prior to exsanguination.

Observations were recorded using a Dictaphone (Olympus VN-713PC, Olympus, Hachioji-shi, Tokyo, Japan) and headset (Sennheiser PC2, Sennheiser electronic GmbH & Co, Wedemark, Germany).

### Electroencephalographic recording and assessment

Electroencephalographic data were recorded using three 27-gauge stainless steel subdermal electrodes (Neuroline Subdermal, Ambu Inc, Glen Burnie, MD, USA), placed as a three-electrode montage in the skin with the: active (non-inverting) right of midline in-line with the back of the eyes; reference (inverting), over the right caudal aspect of the frontal bone (top of head) just rostral of the front of the ears; and ground electrode caudal to the poll on the right. EEG signals were amplified and filtered with an analog filter (Dual Bio Amp, ADInstruments Ltd, Sydney, Australia) with low- and high-pass filters of 100 and 0.1 Hz, respectively. The signals were digitalised (1 kHz) with a 4/35 PowerLab (ADInstruments Ltd, Sydney, Australia) digital-to-analogue converter and recorded on an Apple MacBook Air (Apple Inc Cupertino, CA, USA) for off-line analysis. Interelectrode impedance was tested and ranged between 1.0 and 1.9 kΩ (MkIII Checktrode, UFI, Morro Bay, CA, USA). Each animal acted as their own control with comparisons made between pre- (PRE; 20 s) and post-treatment (POST; 30 s) EEG waveforms. After the completion of EEG recording, the electrodes were removed and the animals ejected from the stunning box, shackled and hoisted and then bled in accordance with routine procedure of the abattoir.

EEG epochs contaminated by artefacts such as over- and under-scale (DC drift), large single spikes, or electromyography were manually rejected from analysis using Chart 8.1.24 (ADInstruments Ltd). All waveforms were digitally filtered with a passband of 1 to 30 Hz, and traces were inspected visually and compared with baseline using the classification systems developed by Gibson *et al.* (2009a,b, 2019). They were classified into one of five categories: (1) movement artefact; (2) normal-like EEG; (3) transitional EEG; (4) high amplitude low frequency (HALF) EEG; and (5) isoelectric EEG. Briefly, normal-like EEG represents an activity that is similar in amplitude and frequency to the baseline period. Transitional EEG was classified as suppressed activity of having either an amplitude of less than half of that of the pre-treatment EEG and/or depressed high-frequency activity. HALF EEG was classified as waveforms of predominantly high amplitude, low frequency activity based on morphology. Isoelectric EEG was classified as a trace with an amplitude of < 1/8 (12.25%) of that of normal-like pre-stunning EEG with little or no low-frequency components.

The EEG power spectra of uncontaminated epochs were analysed. Fast Fourier transformation with a Welch window was applied to non-overlapping 1-s epochs (PRE and every second POST), generating sequential power spectra with 1-Hz frequency bins. Spectral data contaminated by movement artefacts were excluded based on the subjective analysis.

EEG spectral data were calculated and are displayed as percentage changes in total power (Ptot), delta (0.5 to 4 Hz), theta (4 to 8 Hz), alpha (8 to 12 Hz), and beta (12 to 30 Hz) power from PRE values.

### Shot position assessment

Donkeys’ heads were removed (the majority at the atlanto-occiptial joint but some were removed between the atlas [C1] and axis cervical vertebra [C2]) immediately post-bleeding and prior to skinning, and morphometric measurements taken post-skinning. Head length from the top of the poll to the tip of the nasal plane was measured, along with the width of the head from the widest point of each eye and the distance from shot position to the tip of the nasal plane. For each animals, the shot entry position on the cranium was determined as the deviation (lateral and rostro-caudal), from 10 mm above the intersection of lines (determined with placement of durapore tape [3M Company Maplewood, MN, USA]) from the middle of each eye to the base of the opposite ear (Humane Slaughter Association 2016). This position was recorded on transparent acetates placed over the head that was previously skinned.

### Statistical analysis

Data were recorded and entered into Microsoft Excel® 2016 (Microsoft Corporation, Redmond, USA). SPSS (IBM® SPSS Statistics 28.0.0.0, 2022) and R version 4.3.1. (R Core Team 2023) within the RStudio environment were used for statistical analyses. Descriptive behavioural data were reported as proportion (percentage of animals showing behaviour). Distribution of numeric data were tested with the Shapiro-Wilk test. Normally distributed (EEG and shot deviation) data were analysed with paired *t*-test or repeated measures ANOVA to determine associations between variable factors (e.g. shot position/deviation) and outcome (EEG or behavioural signs); while non-normally distributed data were analysed with Mann-Whitney *U* or Friedmann tests, where appropriate. Sagittal and lateral deviations were reported as mean (± SD) and *P* ≤ 0.05 used as the indicator of significance, with trends indicated where *P* < 1.0.

## Results

### Electroencephalography (EEG)

**Figure 1.**
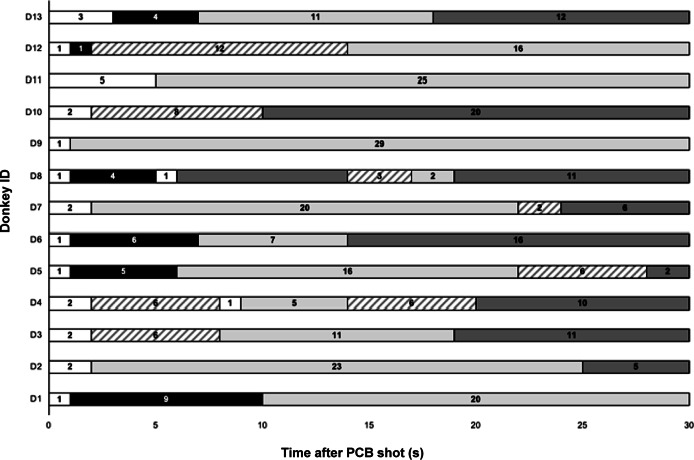
Characteristics of electroencephalogram (EEG) waveforms in individual donkeys (time-point 0) shot with Penetrating Captive Bolt (PCB). White bars represent movement artefact; light grey transitional EEG; dark grey isoelectric EEG; cross-hatched high amplitude low frequency (HALF), and black bars representing normal-like active EEG. Numbers represent duration of the EEG states (s). Figure 1 long description.

Immediately after application of PCB all donkeys had periods of movement artefact in the EEG (Figure 1). The duration varied between donkeys with a mean (± SD) of 1.8 (± 1.1) s; range: 1–5 s. Seven donkeys were classed as completely concussed based on EEG data, in these animals movement artefact was followed by periods of HALF (8.0 [± 9.1]; range: 6–12 s) or transitional (mean duration 18.8 [± 9.1]; range: 11–23 s) EEG activity, before becoming isoelectric in five of the donkeys. The EEG of donkeys D9 and D11 remained transitional during the entire POST 30-s data recording period. No animal received a repeat shot attempt.

Six donkeys (46%; 6/13) had periods of apparently normal-like EEG activity after application of PCB, this period was confined to the initial 10 s post-shooting, before reverting to either HALF or transitional activity. The mean duration of normal-like EEG activity was 4.8 (± 2.6); range: 1–9 s. In four (66%; 4/6) of these animals, the EEG eventually became isoelectric before the end of the data recording period. The EEG of donkeys D1 and D12 was transitional by the end of the 30-s data recording period.

**Figure 2.**
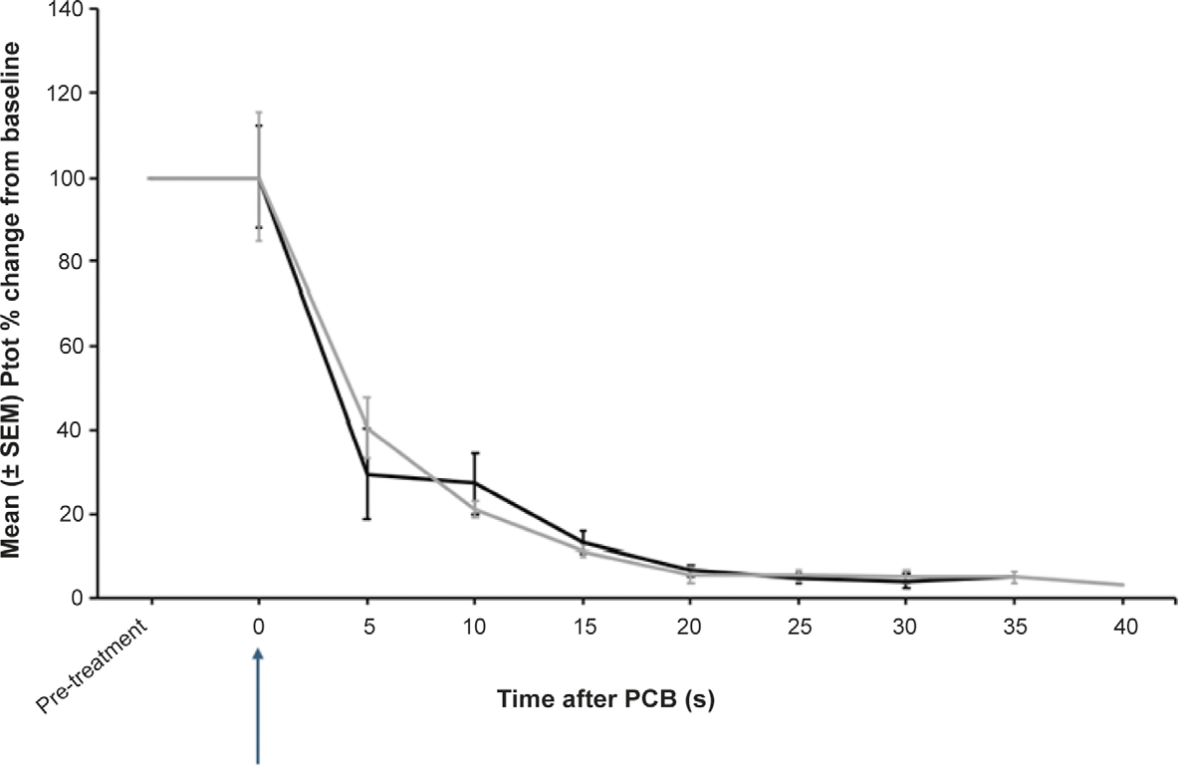
Mean (± SEM) total power (Ptot) of the electroencephalogram (EEG) of donkeys (n = 13) before and after being shot (time-point 0, blue arrow) with a pneumatically powered Penetrating Captive Bolt (PCB) at a Mexican abattoir. The grey line represents donkeys (n = 6) that had periods of incomplete concussion and the black line those that were completely concussed (n = 7). Note this excludes periods of movement artefact for incomplete concussion (time-points 25 s, 35s; n = 5 and 40 s; n = 2) and complete concussion (time-points 5 s; n = 5 and 35; n = 3). Figure 2 long description.

**Figure 3.**
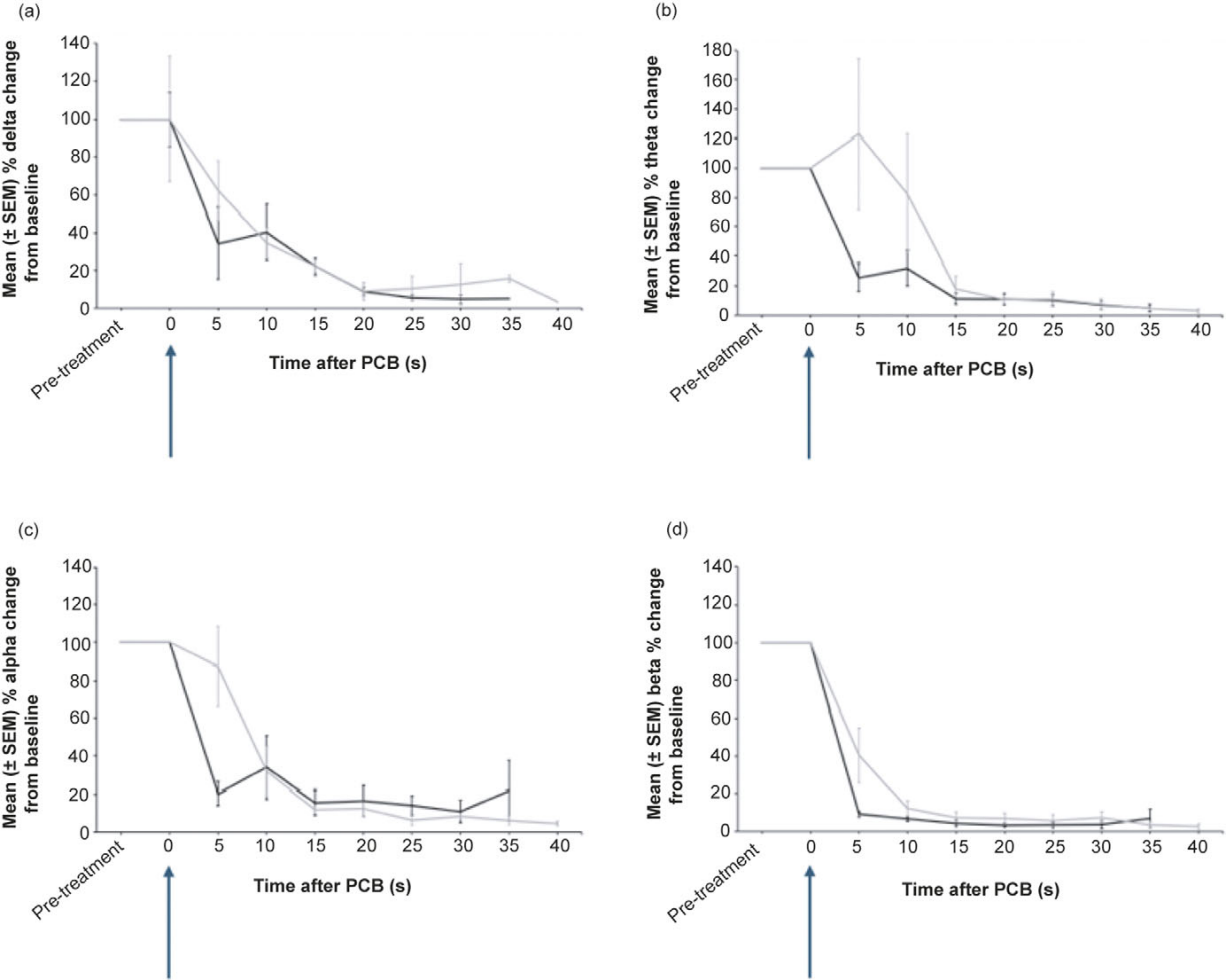
Mean (± SEM) power of (a) delta, (b) theta, (c) alpha and (d) beta frequency bands of the electroencephalogram (EEG) of donkeys (n = 13) before and after being shot (time-point 0, blue arrows) with a pneumatically powered Penetrating Captive Bolt (PCB) at a Mexican abattoir. The grey line represents donkeys that had periods of incomplete concussion (n = 6) and the black line those that were completely concussed (n = 7). Note this excludes periods of movement artefact for incomplete concussion (time-points 25 s, 35s; n = 5 and 40 s; n = 2) and complete concussion (time-points 5 s; n = 5 and 35; n = 3). Figure 3 long description.

After PCB, there was a significant decrease in Ptot, as a percentage change from pre-treatment values (*P* = 0.002) (Figure 2). There was no significant difference in Ptot after 5 s between donkeys that were assessed as incomplete or completely concussed (*P* = 1). In completely concussed donkeys, power in the beta frequency band significantly decreased from pre-treatment values within 5 s of PCB (*P* = 0.01) and remained depressed for the rest of the recording period. For frequency bands delta, theta and alpha there was a non-significant trend towards a decrease (*P* = 0.625) 5 s after PCB. Theta, alpha and beta power remained elevated in the initial 5–10 s after the PCB shot in animals that were incompletely concussed (Figure 3). However, this was momentary before reducing to similar levels as completely concussed animals, with no significant difference between the two groups (completely and incompletely concussed; all *P*-values were *P* > 0.05).

### Shot position

**Figure 4.**
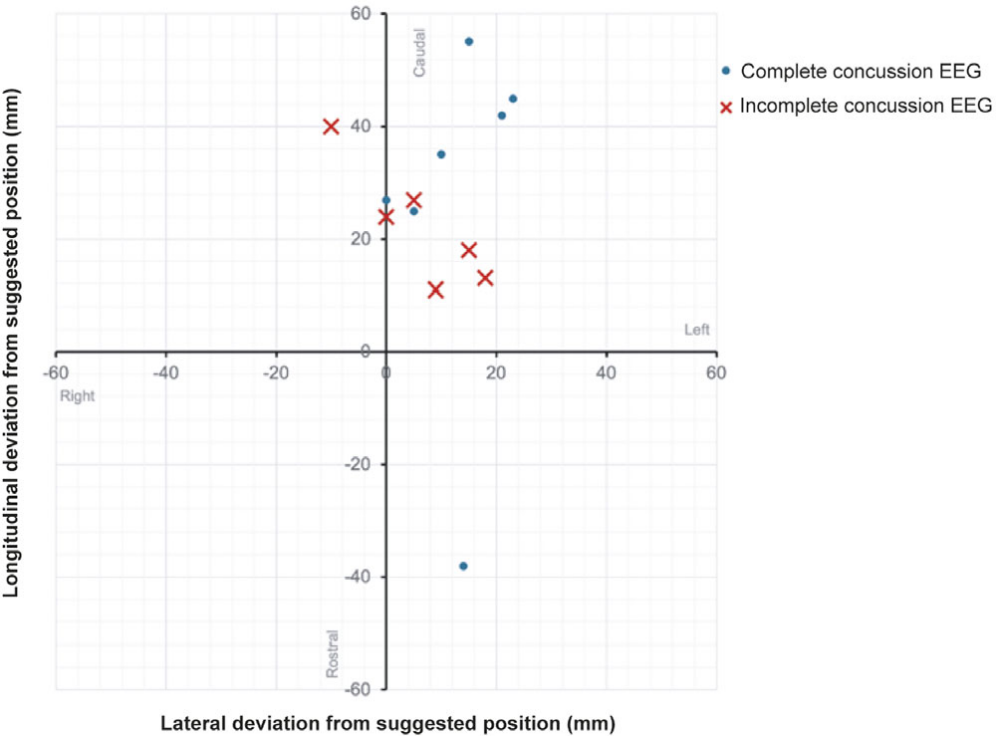
Shot entrance site relative to the Humane Slaughter Association (HSA 2016) suggested position for donkeys (0 mm) shot with a pneumatically powered Penetrating Captive Bolt (PCB) at a Mexican abattoir (n = 13) based on the operator’s perspective (+ is left and caudal of the animal’s midline). Solid blue circles represent donkeys that were completely concussed based on electroencephalogram (EEG) data, while red crosses represent those incompletely concussed based on EEG. Figure 4 long description.

The mean (± SD) deviation from the suggested shot position was 30.8 (± 13.1) mm rostral-caudal and 10.4 (± 6.5) mm lateral (Figure 4), with 92% (12/13) of shots caudal of this position. Mann-Whitney *U* tests found a significant relationship between the presence of normal-like EEG activity post-shot and rostral-caudal deviation (*P* = 0.02) but not lateral deviation (*P* = 0.73). Similarly, there was a significant relationship between behavioural/reflex signs of incomplete concussion and rostral-caudal deviation (*P* = 0.02) but not lateral (*P* = 0.22).

### Behavioural/brainstem signs

One donkey (D6) was classified as incompletely concussed, showing both rhythmic respiration and spontaneous blinking, but was not re-shot (Table 2).

**Table 2.** Behavioural and cranial/spinal responses (%) of donkeys (n = 13) shot with pneumatic penetrating captive bolt (PCB) in a Mexican abattoir Table 2. long description.

Behaviours cranial/spinal responses	Proportion (%)
Immediate collapse	13/13 (100%)
Righting reflex	0/13 (-)
Vocalisations	0/13 (-)
Rhythmic respiration	1/13 (8%)
Gasping	2/13 (15%)
Corneal reflex	0/9* (-)
Palpebral reflex	1/9* (11%)
Spontaneous blinking	2/12* (17%)
Nystagmus	0/13 (-)
Eyeball rotation	0/12* (-)
Response to nostril pinch	0/9* (-)
Involuntary muscle spasms	5/13 (39%)

* Some responses could not be recorded from all animals due to accessibility issues.

D1 had a shallow depth of concussion based on behavioural/brainstem signs, displaying both a palpebral reflex and spontaneous blinking. This donkey also displayed normal-like EEG activity for 9 s post-PCB but was not re-shot. Meanwhile, D5 showed normal-like EEG activity post-shot, was unable to have ocular reflexes checked due to accessibility issues but did show muscle spasms. Similarly, the ocular reflex could not be assessed for donkey D6, but this animal did have a period of normal-like EEG post-PCB and displayed both rhythmic respiration and spontaneous blinking. Donkey D8 had a short period of normal-like EEG activity (4 s) after PCB and was also observed to show gasping post-shot and briefly head shake laterally. Meanwhile, donkeys 12 and 13 had 1- and 4-s periods of normal-like EEG activity, respectively, but presented with no behavioural signs of incomplete concussion. Donkey D10 was the only completely concussed animal (based on EEG data), that did not display any behavioural/reflexes, except for gasping, which was agonal in nature.

## Discussion

This study is the first to examine the EEG responses of donkeys to PCB stunning and explore how these responses related to behavioural signs indicative of brainstem activity. Assessment of EEG is generally considered the most objective, reliable way to assess unconsciousness and predict brain injury prognosis, compared with behavioural indicators (Verhoeven *et al.* 2015; Wang *et al.* 2021). EEG can complement and validate behavioural indicators after stunning to demonstrate the level of consciousness an animal is experiencing, whether they are showing ‘normal-like’ or baseline EEG, indicating consciousness, low-frequency ‘transitional’ EEG, indicating potentially recoverable unconsciousness, or isoelectric EEG, where consciousness is considered unrecoverable and the animal is brain dead (Verhoeven *et al.* 2015; Terlouw *et al.* 2016; Gibson *et al.* 2019; Grandin 2020; Kumar *et al.* 2023).

In the study, no animals were determined to have maintained consciousness for the full duration of the recording period. However, six exhibited transient ‘normal-like’ EEG patterns for up to 9 s post-stunning, which subsequently evolved into transitional or high-amplitude low-frequency (HALF) waveforms. Notably, four animals did not reach an isoelectric EEG state, an indicator of irreversible brain dysfunction during the observation period. This raises concern that these animals could have potentially been at risk of regaining some form of consciousness prior to exsanguination. However, this could not be examined due to the recording period being limited to 30 s post-shot due to the operational requirements of the abattoir and to prevent suffering from recovery by ensuring prompt exsanguination. Together, these findings highlight the potential risks associated with PCB stunning of donkeys, particularly regarding the duration of time during which they might maintain consciousness, and the importance of the reduction of the stun-to-stick interval in donkeys to mitigate the return of consciousness in potentially reversibly stunned animals. Furthermore, this study sheds light on the fact that behavioural signs alone can mean that abattoir operators could fail to recognise the maintenance or recurrence of consciousness in some animals prior to exsanguination.

The Humane Slaughter Association (HSA 2016) suggests that exsanguination should occur a maximum of 60 s post-stun. However, the practicality of this being applied under commercial slaughter conditions, to allow for the time required for animals to be mechanically hoisted post-stun, prior to being bled, requires further research.

In this study, there was a high proportion (46%) of donkeys that had periods of normal-like EEG indicating incomplete concussion. Potential causes for this high failure rate could have been the following:

1. *Shot position and angle of the shot*
  Whilst all operators attempted to shoot frontally rather than a poll shot, donkeys tended to be shot caudally of the suggested position, with this deviation associated with the presence of normal-like EEG activity post-stun but not with behavioural signs of ineffective stunning. A caudal shot, combined with a shallow angle due to the fixed-angle of the pneumatic PCB stunner, particularly where donkeys moved or lowered their heads, is more likely to have missed the ascending reticular activating system (ARAS), which regulates cortical activity and conscious awareness. The ARAS includes the thalamus, midbrain and/or rostral pons, with these therefore the target structures for PCB stunning to achieve irrecoverable unconsciousness (Wedekind *et al.* 2009; Terlouw 2020; Edlow *et al* 2021; Fletcher *et al*. 2025). A caudal shot could potentially cause merely superficial damage to the cerebellum, which controls posture and movement, resulting in the animal maintaining or recovering consciousness, but being unable to right itself (Grist *et al.* 2019; Terlouw 2020; Večerek *et al.* 2020; Gibson *et al.* 2025). There is also a risk of the shot hitting the spinal cord, causing paralysis but not irrecoverable unconsciousness (Terlouw 2020). However, this hypothesis could not be verified, as brain pathology was not performed on these donkeys.
2. *Restraint*
  In this study, the donkeys were restrained by a neck rope held tightly by personnel, enabling more precise application of the EEG electrodes and consequently a more precise shot to the suggested position on the head. However, head restraint is not typically used for equids being slaughtered by PCB (Fletcher *et al.* 2024) and was not usual practice for this abattoir, potentially impacting shot position and causing additional stress to the donkey prior to stunning. Restraint and placement of the PCB were complicated by the design of the stunning box, which was initially designed for cattle, and operators were required to lean down into the box to reach the donkeys, who were much smaller in stature than slaughter-weight cattle.
3. *Airline pressure and velocity*
  Stunning effectiveness could also have been influenced by airline pressure and velocity, although it was not possible to assess and control this. Abattoir operators should check the device regularly to ensure that it is working correctly and capable of reaching an adequate penetration depth to achieve irreversible unconsciousness (Gibson *et al.* 2014; Kamenik et al. 2019; Baier & Willson 2020; Grist *et al.* 2020).
4. *Level of operator experience and training*
  These could also have influenced stunning effectiveness however were unable to be verified due to a lack of information on training levels of expertise.
5. *Lack of specific guidance for stunning position for donkeys*
  Use of the same reference points suggested for horses is commonplace and was the case in this study. However, even if similar positioning could be hypothesised, this does not account for differences in skull morphology between these two species (Merkies *et al.* 2020). This could have partially influenced the number of ineffectively stunned animals. For this reason, empirical anatomical studies on appropriate shooting and stunning position for donkeys would be valuable further research.

Out of the six animals that showed periods of ‘normal like’ EEG, only one based on behavioural/brainstem signs, was classified as incompletely concussed, four showed behavioural signs and reflexes potentially indicating signs of a shallow depth of concussion, while the other two did not show any signs. Brain pathology could have confirmed whether these two animals which were not showing signs, had any marked trauma to key brain regions. This highlights how reliance on behavioural signs alone may not be reflective of the actual experience of the animal, potentially resulting in the underestimation of the animals that are still conscious after stunning. Whilst animals should always be routinely monitored between stunning and bleeding, an absence of behavioural signs does not absolutely ensure unconsciousness. This is particularly relevant where animals have incurred a shot into the spinal cord, absence of behavioural signs can mean paralysis rather than unconsciousness (Kumru *et al.* 2010; Terlouw *et al.* 2021; Fletcher *et al.* 2025). However, despite most shots being caudal of the suggested position, potentially into the occipital area, there was no attempt in the present study to shoot animals into the poll (behind the ears) rather than frontally (rostral of the ears), which would have increased the risk of such spinal cord paralysis.

Two animals displayed gasping, although for D10, this is likely to have been agonal in nature due to cerebral hypoxia, and the absence of EEG and behavioural signs of incomplete concussion. Gasping has been reported as a parameter of risk of consciousness or return to consciousness, however it can also be observed in unconscious states (Terlouw *et al.* 2021). Gasping is considered the final respiratory effort to sustain life (Poets *et al.* 1999). It is generated by an intrinsic medullary mechanism that is recruited when there is a dysfunction in the pons (St John 2009). Gasping corresponds to intermittent, inspiratory movements with an open mouth which can be induced by ischaemia or hypoxia (St John 2009). Gasping is very different from rhythmic breathing, and it is often accompanied by guttural sounds (Terlouw *et al.* 2016). Blackmore and Newhook (1982) reported a PCB-shot calf displaying non-rhythmic gasping after having an isoelectric EEG for 60 s. Together, this suggests that although gasping can be a sign of the early stages of return of consciousness, it should be interpreted with caution and not in isolation from other indices (Terlouw *et al.* 2021). It is therefore vital that multiple indices are used to determine shot effectiveness, rather than focusing on a single measure. Tools such as EEG are impractical for use in routine commercial slaughter situations, with abattoir operatives therefore needing to rely on behavioural and brainstem indices as important routine measures, despite their limitations, but still exercising caution even where no responses are observed (Terlouw *et al.* 2016; Terlouw 2020; Fletcher *et al*. 2025).

The single animal that appeared to show more overt rhythmic respiration, also showed spontaneous blinking and a period of normal-like EEG. If just rhythmic respiration had been seen in the absence of other indices, this may not necessarily indicate consciousness, but suggests that, particularly alongside the presence of eye reflexes, the animal might be more likely to recover consciousness through oxygenated blood continuing to reach the brain (Terlouw *et al.* 2016; Borzuta *et al.* 2019).

### Study limitations

Although this study provided the first insights into stunning effectiveness in donkeys, the results must be interpreted with caution as there are certain limitations. Firstly, the sample size of 13 donkeys was small, but unavoidable due to the complexity of abattoir-based EEG research. Secondly, brain pathology of the slaughtered donkeys was not conducted, macro- or microscopically, to determine the precise location of the shot alongside the level and extent of brain trauma, with stunning position measured on skinned heads. This was unavoidable due to the field-based working conditions. Thirdly, animals were not monitored between hoisting and exsanguination, which was not possible due to the design of the abattoir and the risk it presented to researcher safety. The study was also observational in nature, with no interference by the research team on commercial decision making, including shot effectiveness and re-stunning.

In some studies, local anaesthesia is used to desensitise the skin prior to EEG electrode placement (Gibson *et al.* 2018; Rucinque *et al.* 2024). However, in this study it was not possible to use either topical ELMA cream or injected local anaesthetic due to the additional time and stress further handling would cause, and importantly because these were food-producing animals. EMLA cream is not licensed for food-producing animals, and if prescribed would have required withdrawal period (28 days in the UK).

Whilst EEG assessment post-stun briefly delayed exsanguination by a few seconds, abattoir personnel should be routinely monitoring animals from stun to stick, repeating the shot if there is any doubt of effectiveness, and generally minimising delays between these stages to ensure rapid exsanguination. This should then minimise the risk of animals recovering during this period. Finally, the presence of researchers could have influenced both operator and animal behaviour. However, the research team were cognisant to not interfere with the operation of the abattoir, and these risks tend to be unavoidable in commercial conditions.

## Animal welfare implications and Conclusion

This study provides the first insight into electroencephalographic (EEG) responses of donkeys following penetrating captive-bolt (PCB) stunning. While PCB stunning generally induced insensibility, the presence of transient normal-like EEG patterns and the absence of isoelectric signals in some animals raise critical animal welfare concerns. These findings suggest a risk of delayed or incomplete loss of consciousness, or recovery of consciousness prior to exsanguination, particularly when shot placement deviates from the optimal trajectory or when stun-to-stick intervals are prolonged. The study emphasises the need for species-specific guidelines for donkeys, improvements in abattoir design, and stricter monitoring protocols to ensure effective stunning and minimise the risk of recovery, pain or distress during slaughter.

## Table 1. Long description

From the top row downward, the left column lists behaviours and cranial or spinal responses, while the right column provides descriptions. The entries are: Lack of immediate collapse, described as the animal failing to collapse immediately after the shot; Righting reflex, making a coordinated effort to stand or lift head; Vocalisations, vocalising independently from exhalation; Rhythmic respiration, ribcage moving in and out rhythmically; Gasping, spasmodic sharp intake of breath with mouth open; Corneal reflex, involuntary blink reflex when cornea is stimulated; Palpebral reflex, involuntary blink reflex when the medial canthus is stimulated; Spontaneous blinking, opening or closing eyelid without stimulation; Nystagmus, rapid involuntary movements of the eye; Eyeball rotation, eyes rotated, not central, sclera visible; Response to nostril pinch, pinching nasal septum followed by pain reaction or withdrawal; Involuntary muscle spasms, absence of tonus in body or excessive muscle activity.

Navigate back to Table 1..

## Figure 1 Long description

Horizontally stacked barchart showing characteristics of electroencephalogram (EEG) waveforms in individual donkeys (time- point 0) shot with Penetrating Captive Bolt (PCB). White bars represent movement artefact; light grey transitional EEG; dark grey isoelectric EEG; cross- hatched high amplitude low frequency (HALF), and black bars representing normal-like active EEG. Numbers represent duration of the EEG states (s).

Navigate back to Figure 1.

## Figure 2 Long description

Line graph showing mean (± SEM) total power (Ptot) of the electroencephalogram (EEG) of donkeys (n = 13) before and after being shot (time- point 0, blue arrow) with a pneumatically powered Penetrating Captive Bolt (PCB) at a Mexican abattoir (n = 13). The grey line represents donkeys (n = 6) that had periods of incomplete concussion (n = 6) and the black line those that were completely concussed (n = 7). Note this excludes periods of movement artefact for incomplete concussion (time-points 25 s, 35s; n = 5 and 40 s; n = 2) and complete concussion (time-points 5 s; n = 5 and 35; n = 3).

Navigate back to Figure 2.

## Figure 3 Long description

Line graph showing mean (± SEM) power of delta (A), theta (B), alpha (C) and beta (D) frequency bands of the electroencephalogram (EEG) of donkeys (n = 13) before and after being shot (time-point 0, blue arrows) with a pneumatically powered Penetrating Captive Bolt (PCB) at a Mexican abattoir (n = 13). The grey line represents donkeys that had periods of incomplete concussion (n = 6) and the black line those that were completely concussed (n = 7). Note this excludes periods of movement artefact for incomplete concussion (time--points 25 s, 35s; n = 5 and 40 s; n = 2) and complete concussion (time-points 5 s; n = 5 and 35; n = 3).

Navigate back to Figure 3.

## Figure 4 Long description

Scatterplot showing shot entrance site relative to the Humane Slaughter Association (HSA 2016) suggested position for donkeys (0 mm) shot with a pneumatically powered Penetrating Captive Bolt (PCB) at a Mexican abattoir (n = 13) based on the operator’s perspective (+ is left and caudal of the animal’s midline). Solid blue circles represent donkeys that were completely concussed based on electroencephalogram (EEG) data, while red crosses represent those incompletely concussed based on EEG.

Navigate back to Figure 4.

## Table 2. Long description

Table outlining behavioural and cranial/spinal responses of donkeys (%) shot with pneumatic penetrating captive bolt (PCB) (n=13) in a Mexican abattoir.

Navigate back to Table 2..

## References

[r1] Ashley FH, Waterman-Pearson AE and Whay HR 2005 Behavioural assessment of pain in horses and donkeys: application to clinical practice and future studies. Equine Veterinary Journal 37(6): 565–575. 10.2746/042516405775314826 16295937

[r2] Baier F and Willson D 2020 Basics of captive bolt stunning of cattle and other animals. The Slaughter of Farmed Animals: Practical Ways of Enhancing Animal Welfare pp 145–158. CABI: Wallingford, UK. 10.1079/9781789240573.0145

[r3] Bennett R and Pfuderer S 2019 Demand for donkey hides and implications for global donkey populations. *Proceedings of the 93rd Annual Conference.* 15–17 April 2019, Coventry, UK.

[r4] Blackmore DK and Newhook JC 1982 Electroencephalographic studies of stunning and slaughter of sheep and calves—Part 3: The duration of insensibility induced by electrical stunning in sheep and calves. Meat Science 7(1) 19–28. 10.1016/0309-1740(82)90094-8 22055065

[r5] Borzuta K, Lisiak D, Janiszewski P and Grześkowiak E 2019 The physiological aspects, technique and monitoring of slaughter procedures and their effects on meat quality – a review. Annals of Animal Science 19(4): 857–873. 10.2478/aoas-2019-0039

[r6] Dalla Costa FA, Gibson TJ, Oliveira SEO, Gregory NG, Coldebella A, Faucitano L, Ludtke CB, Peréirã Buss L and Dalla Costa OA 2020 Evaluation of physical euthanasia for neonatal piglets on-farm. Journal of Animal Science 98(7): skaa204. 10.1093/jas/skaa204 32620008 PMC7455299

[r7] Edlow BL, Claassen J, Schiff ND and Greer DM 2021 Recovery from disorders of consciousness: mechanisms, prognosis and emerging therapies. Nature Reviews: Neurology 17: 135–156. 10.1038/s41582-020-00428-x 33318675 PMC7734616

[r8] Fletcher K, Benedetti B, Limon G, Grist A, Padalino B, Hernández Gil M and Gibson T J 2025 Pathophysiology of penetrating Captive Bolt Gun stunning of horses. Animal Welfare 34: e51. 10.1017/awf.2025.10025 40735425 PMC12304776

[r9] Fletcher K, Limon G, Agongo E, Akunzule A, Essel G, Padalino B, Grist A and Gibson TJ 2024 Assessment of Donkey ( *Equus asinus* ) Welfare at Slaughter in Ghana. Animals 14: 3673. 10.3390/ani14243673 39765577 PMC11672695

[r10] Fletcher KA, Limon G, Whatford LJ, Grist A, Knowles TG and Gibson TJ 2022 A systematic review of equid welfare at slaughter. Livestock Science 263: 104988. 10.1016/J.LIVSCI.2022.104988

[r11] Gameiro MBP, Rezende VT and Zanella AJ 2021 Brazilian donkey slaughter and exports from 2002 to 2019. Brazilian Journal of Veterinary Research and Animal Science 58: e174697. 10.11606/issn.1678-4456.bjvras.2021.174697

[r12] Gibson TJ, Bedford EM, Chancellor NM and Limon G 2015 Pathophysiology of free-bullet slaughter of horses and ponies. Meat Science 108: 120–124. 10.1016/j.meatsci.2015.06.007 26093383

[r13] Gibson TJ, Rebelo RB, Gowers TA, and Chancellor N 2018 Electroencephalographic assessment of concussive non-penetrative captive-bolt stunning of turkeys. British Poultry Science 59(1): 13–20. 10.1080/00071668.2017.1401215 29099622

[r14] Gibson TJ, Ridler AL, Limon G, Lamb C, Williams A and Gregory NG 2025 Pathophysiology of Penetrating Captive Bolt Stunning in Horned and Polled Sheep and Factors Determining Incomplete Concussion. *Veterinary Sciences*; 12(1): 53. 10.3390/vetsci12010053 39852928 PMC11769382

[r15] Gibson TJ, Johnson CB, Murrell JC, Mitchinson SL, Stafford KJ and Mellor DJ 2009a Electroencephalographic responses to concussive non-penetrative captive-bolt stunning in halothane-anaesthetised calves. New Zealand Veterinary Journal 57: 90–95. 10.1080/00480169.2009.36884 19471327

[r16] Gibson TJ, Johnson CB, Murrell JC, Hulls CM, Mitchinson SL, Stafford KJ, Johnstone AC and Mellor DJ 2009b Electroencephalographic responses of halothane-anaesthetised calves to slaughter by ventral-neck incision without prior stunning. New Zealand Veterinary Journal 57: 77–83. 10.1080/00480169.2009.36882 19471325

[r17] Gibson TJ, Mason CW, Spence JY, Barker H and Gregory NG 2014 Factors Affecting Penetrating Captive Bolt Gun Performance, Journal of Applied Animal Welfare Science 18(3): 222–238. 10.1080/10888705.2014.980579 25415241

[r18] Gibson TJ, Oliveira SEO, Dalla Costa FA and Gregory NG 2019 Electroencephalographic assessment of pneumatically powered penetrating and non-penetrating captive-bolt stunning of bulls. Meat Science 151: 54–59. 10.1016/j.meatsci.2019.01.006 30685511

[r19] Grandin T, 2020 Determining unconsciousness and insensibility in commercial abattoirs. In: Grandin, T. and M. Cockram, editors. The slaughter of farmed animals: practical ways of enhancing animal welfare. Oxfordshire (UK): CAB International; p. 193–201.

[r20] Grist A, Bock R, Knowles TG and Wotton S B 2020 Further examination of the performance of blank cartridges used in captive bolt devices for the pre-slaughter stunning of animals. Animals 10(11): 1–12. 10.3390/ani10112146 PMC7698744 33218032

[r21] Grist A, Knowles T G and Wotton S 2019 Macroscopic examination of multiple-shot cattle heads—an animal welfare due diligence tool for abattoirs using penetrating captive bolt devices? Animals 9(6): 328. 10.3390/ani9060328 31174418 PMC6616863

[r22] Humane Slaughter Association 2016 *Captive-Bolt Stunning of Livestock.* www.hsa.org.uk

[r23] Kamenik J, Paral V, Pyszko M and Voslarova E 2019 Cattle stunning with a penetrative captive bolt device: A review. Animal Science Journal 90(3): 307–316. Blackwell Publishing: London, UK. 10.1111/asj.13168 30669179

[r24] Kumar P, Abubakar AA, Imlan JC, Ahmed MA, Goh YM, Kaka U, Idrus Z and Sazili AQ 2023 Importance of Knife Sharpness during Slaughter: Shariah and Kosher Perspective and Scientific Validation. Animals 13: 1751. 10.3390/ani13111751 37889669 PMC10251950

[r25] Kumru H, Vidal J, Kofler M, Portell E and Valls-Solé J 2010 Alterations in Excitatory and Inhibitory Brainstem Interneuronal Circuits after Severe Spinal Cord Injury. Journal of Neurotrauma 27(4): 721–728. 10.1089/NEU.2009.1089 20067395

[r26] Merkies K, Paraschou G and McGreevy PD 2020 Morphometric Characteristics of the Skull in Horses and Donkeys—A Pilot Study. Animals 10(6): 1002. 10.3390/ani10061002 32521777 PMC7341236

[r27] Norris SL, Little HA, Ryding J and Raw Z 2021 Global donkey and mule populations: Figures and trends. PLoS ONE 16: e0247830. 10.1371/journal.pone.0247830 33630957 PMC7906361

[r28] Oliveira SEO, Dalla Costa FA, Gibson TJ, Dalla Costa OA, Coldebella A and Gregory NG 2018 Evaluation of brain damage resulting from penetrating and non–penetrating stunning in Nelore Cattle using pneumatically powered captive bolt guns. Meat Science 145: 347–351. 10.1016/j.meatsci.2018.07.016 30029088

[r29] Poets CF, Meny RG, Chobanian MR and Bonofiglo RE 1999 Gasping and other cardiorespiratory patterns during sudden infant deaths. Pediatric Research 45(3): 350–354. 10.1203/00006450-199903000-00010 10088653

[r30] R Core Team 2023 R: A language and environment for statistical computing. R Foundation for Statistical Computing, Vienna, Austria. https://www.R-project.org/.

[r31] Rucinque DS, Velarde A, Xercavins A, Varvaró-Porter A, Gibson TJ, Michel V and Contreras-Jodar A 2024 Alternatives to the use of carbon dioxide in two phases for the stunning of broiler chickens at slaughter. Animals 14 (486). 10.3390/ani14030486 PMC10854911 38338133

[r32] St John WM 2009 Noeud vital for breathing in the brainstem: Gasping—yes, eupnoea—doubtful. Philosophical Transactions of the Royal Society B 364: 2625–2633. 10.1098/rstb.2009.0080 PMC2865115 19651662

[r33] Terlouw C 2020 The physiology of the brain and determining insensibility and unconsciousness. *The Slaughter of Farmed Animals: Practical Ways of Enhancing Animal Welfare* pp 202–228. 10.1079/9781789240573.0202

[r34] Terlouw C, Bourguet C and Deiss V 2016 Consciousness, unconsciousness and death in the context of slaughter. Part II. Evaluation methods. Meat Science 118: 147–156, 10.1016/j.meatsci.2016.03.010 27086068

[r35] Terlouw C, Ducreux B and Bourguet C 2021 Specificities of consciousness and unconsciousness indicators according to slaughter methods. *French Review of Research in Meat and Meat Products*. https://www.viandesetproduitscarnes.com/index.php/bien-etre-animal/99-specificites-des-indicateurs-de-conscience-et-dinconscience-selon-les-methodes-dabattage (accessed 29 April 2026).

[r36] Vecerek V, Kamenik J, Voslarova E, Vecerkova L, Machovcova Z, Volfova M and Konvalinova J 2020 The occurrence of reflexes and reactions in cattle following stunning with a captive bolt at the slaughterhouse. Animal Science Journal 91(1): e13373. 10.1111/asj.13373 32301197

[r37] Verhoeven MTW, Gerritzen MA, Hellebrekers LJ and Kemp B 2015 Indicators used in livestock to assess unconsciousness after stunning: a review. Animal 9(2): 320–330. 10.1017/S1751731114002596 25354537 PMC4299535

[r38] Verhoeven MTW, Gerritzen MA, Hellebrekers LJ and Kemp B 2016 Validation of indicators used to assess unconsciousness in veal calves at slaughter. Animal 10(9): 1457–1465. 10.1017/S1751731116000422 26965337

[r39] Wang J, Huang L, Ma X, Zhao C, Liu J and Xu D 2021 Role of Quantitative EEG and EEG Reactivity in Traumatic Brain Injury. Clinical EEG and Neuroscience 53(5): 452–459. 10.1177/1550059420984934 33405972

[r40] Wedekind C, Hesselmann V, Lippert-Grüner M and Ebel M 2009 Trauma to the pontomesencephalic brainstem: a major clue to the prognosis of severe traumatic brain injury. British Journal of Neurosurgery 16(3): 256–260. 10.1080/02688690220148842 12201395

[r41] Zulkifli I, Goh YM, Norbaiyah B, Sazili AQ, Lotfi M, Soleimani AF and Small AH 2014 Changes in blood parameters and electroencephalogram of cattle as affected by different stunning and slaughter methods in cattle. Animal Production Science 54: 187–193. 10.1071/AN12128

